# Comparison of the Pulmonary Oxidative Stress Caused by Intratracheal Instillation and Inhalation of NiO Nanoparticles when Equivalent Amounts of NiO Are Retained in the Lung

**DOI:** 10.3390/antiox5010004

**Published:** 2016-01-18

**Authors:** Masanori Horie, Yukiko Yoshiura, Hiroto Izumi, Takako Oyabu, Taisuke Tomonaga, Takami Okada, Byeong-Woo Lee, Toshihiko Myojo, Masaru Kubo, Manabu Shimada, Yasuo Morimoto

**Affiliations:** 1 Health Research Institute (HRI), National Institute of Advanced Industrial Science and Technology (AIST), 2217-14 Hayashi-cho, Takamatsu 761-0301, Kagawa, Japan; 2 Institute of Industrial Ecological Sciences, University of Occupational and Environmental Health, 1-1, Iseigaoka, Yahata-nishi-ku, Kitakyushu, Fukuoka 807-8555, Japan; y-yoshiura@med.uoeh-u.ac.jp (Y.Y.); h-izumi@med.uoeh-u.ac.jp (H.I.); toyabu@med.uoeh-u.ac.jp (T.O.); t-tomonaga@med.uoeh-u.ac.jp (T.T.); okadatakami@med.uoeh-u.ac.jp (T.O.); leebw401@med.uoeh-u.ac.jp (B.-W.L.); tmyojo@med.uoeh-u.ac.jp (T.M.); yasuom@med.uoeh-u.ac.jp (Y.M.); 3 Department of Chemical Engineering, Hiroshima University, 1-4-1 Kagamiyama, Higashi-Hiroshima, Hiroshima 739-8527, Japan; mkubo@hiroshima-u.ac.jp (M.K.); smd@hiroshima-u.ac.jp (M.S.)

**Keywords:** oxidative stress, nanoparticle, nickel oxide, inhalation, intratracheal instillation

## Abstract

NiO nanoparticles were administered to rat lungs via intratracheal instillation or inhalation. During pulmonary toxicity caused by NiO nanoparticles, the induction of oxidative stress is a major factor. Both intratracheal instillation and inhalation of NiO nanoparticles induced pulmonary oxidative stress. The oxidative stress response protein, heme oxygenase-1 (HO-1), was induced by the administration of NiO nanoparticles at both the protein and gene expression level. Additionally, certain oxidative-stress markers in the lung, such as 8-iso-prostaglandin F2α, thioredoxin, and inducible nitric oxide synthase were increased. Furthermore, the concentration of myeloperoxidase (MPO) in the lung was also increased by the administration of NiO nanoparticles. When the amount of NiO in the lung is similar, the responses against pulmonary oxidative stress of intratracheal instillation and inhalation are also similar. However, the state of pulmonary oxidative stress in the early phase was different between intratracheal instillation and inhalation, even if the amount of NiO in the lung was similar. Inhalation causes milder oxidative stress than that caused by intratracheal instillation. On evaluation of the nanoparticle-induced pulmonary oxidative stress in the early phase, we should understand the different states of oxidative stress induced by intratracheal instillation and inhalation.

## 1. Introduction

Evaluation of the pulmonary toxicity caused by nanoparticles is very important for the industrial application of nanoparticles. Intratracheal instillation and inhalation exposure to rodents is frequently employed to evaluate the pulmonary toxicity of nanoparticles. In the latter, animals in exposure chambers inhale an aerosol of nanoparticles by natural aspiration. Thus, inhalation exposure is the most suitable model for nanoparticle exposure in humans. On the other hand, there are downsides to this method; for example, control of the exposure concentration is difficult. In addition, the apparatus required for inhalation exposure, such as an exposure chamber system, is expensive and thus the potential number of subjects is limited. Therefore, intratracheal instillation is frequently employed as an alternative method of inhalation. It has some merits compared with inhalation exposure [1]. Control of dosage is easy and there is certainty that the administrated dose has reached the lung. Many examinations are possible in a short time; it is an effective method for the pre-screening process of an inhalation examination. On the other hand, intratracheal instillation also has disadvantages. It can lead to overload of the material. In some cases, compared with the inhalation test, the dosage with intratracheal instillation is larger. Overload of the materials causes artificial and unphysiological results. These results are not significant for toxicological testing purposes. It is also notable that the distribution of test materials in the respiratory tract is different between inhalation and intratracheal instillation [1]. Administrated via intratracheal injection, the test materials avoid the upper air passage (nasal cavity, mouth, pharynx, and larynx. However, the upper air passage is one of the important targets of inhalation exposure. Distribution of test materials in the lung by intratracheal instillation tends to be uneven [2]. Comparison of inhalation exposure and intratracheal instillation is essential to evaluate whether the latter is effective as an alternative method of inhalation. However, although there are many studies evaluating the pulmonary toxicity of chemicals that individually employ the intratracheal instillation or inhalation methods, few studies compare the two. Damon *et al.* [3] compared the pulmonary toxicity of Triton X-100 caused by intratracheal instillation and inhalation. Although the dose-related lethality (LC50) of intratracheal instillation and inhalation was similar, the dose response curves for each were different. According to the results of a comparison of intratracheal instillation and inhalation of glass fibers, multiple instillations at a low dose showed a similar response to inhalation; however, single instillation led to the possibility of artificial granuloma formation [4]. Driscoll *et al.* compared the inflammation responses and histopathology caused by intratracheal instillation or inhalation of crystalline silica [5,6,7]. The type of responses were similar with intratracheal instillation and inhalation; however, the time course and strength of the responses were different. Generally, biological responses induced by inhalation are slower and weaker than those induced by intratracheal instillation. If the pulmonary responses to intratracheal instillation and inhalation are compared, the retained amount of the test materials in the lung is very important. It is important that the pulmonary response be compared with the same retained amount of test materials in the lung. We previously reported a comparison of the pulmonary toxicity of NiO nanoparticles caused by intratracheal instillation or inhalation with the same retained amount of NiO in the lung [8]. When the retained amount of NiO in the lung was similar, the pulmonary inflammation and injury caused by NiO nanoparticles was also similar with both intratracheal instillation and inhalation. In particular, both intratracheal instillation and inhalation of NiO nanoparticles to rats induced pulmonary inflammation and injury. Metal ion release and the induction of oxidative stress are involved in the pulmonary toxicity caused by toxic nanoparticles such as NiO and ZnO [9,10,11]. Oxidative stress might be related to the pulmonary inflammation caused by NiO nanoparticles. However, there is no report comparing the induction of oxidative stress caused by intratracheal instillation with that caused by inhalation. In the present study, we compared the pulmonary oxidative stress caused by intratracheal instillation or inhalation of NiO nanoparticles, with a similar amount of NiO retained in the lung.

## 2. Experimental Section

### 2.1. NiO Nanoparticles

NiO nanoparticles were purchased from US Research Nanomaterials, Inc. (Houston, TX, USA) (Product code US3355). According to the manufacturer’s data, the primary particle size, specific surface area, and purity were 15–35 nm, 50–100 m^2^/g, and > 99.5% respectively. The color was black. The preparation and characterization of the NiO nanoparticle dispersion for intratracheal instillation and inhalation have been reported previously [8].

### 2.2. Animals

Male Fischer 344 (F344) rats (12 or 10 weeks old for intratracheal instillation or inhalation, respectively) were purchased from Charles River Laboratories Japan, Inc. (Yokohama, Japan). The animals were kept in the Laboratory Animal Research Center of the University of Occupational and Environmental Health for a week with free access to commercial diet and water. All animal experiments were approved by the Institutional Animal Care and Use Committee (IACUC) of the University of Occupational and Environmental Health, Japan. All procedures and animal handling were performed according to the guidelines described in the Japanese Guide for the Care and Use of Laboratory Animals.

### 2.3. Intratracheal Instillation

Either 0.2 mg or 1.0 mg of NiO nanoparticles was dispersed in 0.4 mL of distilled water. Each material dispersion was intratracheally instilled once in rats (12 weeks old). The negative control group was exposed to distilled water. Animals were dissected at 3 days, 1 month, 3 months, and 6 months after instillation. Each group consisted of 10 animals and was divided into two subgroups for lung-tissue analysis. The first subgroup (5 rats) was subjected to bronchoalveolar lavage, which was performed by injecting physiological saline through a cannula inserted in the respiratory tract. Physiological saline (20 mL) was infused into the lung by gravity, and approximately 15 mL lavage fluid was collected in total. The lungs of the second subgroup (5 rats) were homogenized to extract proteins and mRNA.

### 2.4. Inhalation

The generation and the characterization of NiO aerosol nanoparticles and development of an inhalation system for the same are described in our previous paper [12]. The concentration of the particles was 1.65 ± 0.20 mg/m^3^ and 0.32 ± 0.07 mg/m^3^ in the high and low dose chambers, respectively [8]. For inhalation exposure, 10-week-old rats were exposed to NiO nanoparticles in a whole-body exposure chamber (volume, 0.52 m^3^) for 6 h/day and 5 days/week for 4 weeks. The control rats were exposed to clean air in an equivalent-sized chamber located in the same air-conditioned room. The rats were dissected 3 days, 1 month, 3 months, and 6 months after the 4-week exposure period.

### 2.5. Measurement of Oxidative Stress-Related Proteins

The proteins were extracted from the lung tissue. For protein extraction, 50–60 μg of lung tissue was added to 500 μL of T-PER tissue protein extraction reagent (Thermo Scientific Inc., Rockford, IL, USA) including protein inhibitor cocktails (P8340, Sigma-Aldrich, St. Louis, MO, USA) and cOmplete, Mini (Roche Diagnostics, Basel, Switzerland). The lung was homogenized using a homogenizer. Subsequently, the homogenate was centrifuged at 15000 rpm for 10 min. The supernatant was collected and the protein concentration was measured using the Bradford method and the Quick Start Bradford protein assay (Bio-Rad Laboratories, Inc., Hercules, CA, USA). The protein concentration was prepared to 100–2000, 250, and 50 μg/mL for measurement of heme oxygenase-1 (HO-1), thioredoxin (Trx), and myeloperoxidase (MPO), respectively. The HO-1, Trx, and MPO concentrations in the lung were measured using enzyme-linked immunosorbent assay (ELISA) kits for HO-1 (rat; Enzo Life Sciences, Inc., Farmingdale, NY, USA), Trx (Uscn Life Science Inc. Wuhan, China), and MPO (rat; Hycult Biotech Inc., Plymouth Meeting, PA, USA), respectively. The 8-iso-prostaglandin F2α (8-isoPGF2α) in the bronchoalveolar lavage fluid (BALF) was measured using an 8-iso-PGF2α ELISA kit (Enzo Life Sciences).

### 2.6. Measurement of Gene Expression

Gene expression was examined by real-time polymerase chain reaction (PCR). The lung was immediately soaked in RNAlater^®^ RNA stabilization reagent (Qiagen, Hilden, Germany). Lung tissue was homogenized using the TissueRuptor (Qiagen) and then total RNA was prepared using the RNeasy^®^ Mini kit (Qiagen). Real time-PCR was conducted using a Step One real time-PCR system (Applied Biosystems, Life Technologies Japan, Tokyo, Japan), and PCR amplification of the lung tissue was analyzed using TaqMan^®^ gene expression assays (Life Technologies, Thermo Fisher Scientific Inc., Carlsbad, CA, USA), with the rat β-actin gene as an endogenous control. The codes of the probes for the TaqMan^®^ gene expression assays for HO-1, metallothionein (MT) 1A, MT2A, and nitric oxide synthase 2 (Nos2) were Rn00561387_m1, Rn00821759_g1, Rn01536589_g1, and Rn00561646_m1, respectively. The TaqMan^®^ probe for β-actin was purchased from Life Technologies Japan (Code No. 4352340E).

## 3. Results and Discussion

### 3.1. HO-1 Protein and Gene Expression in the Lung Tissue

**Figure 1:**
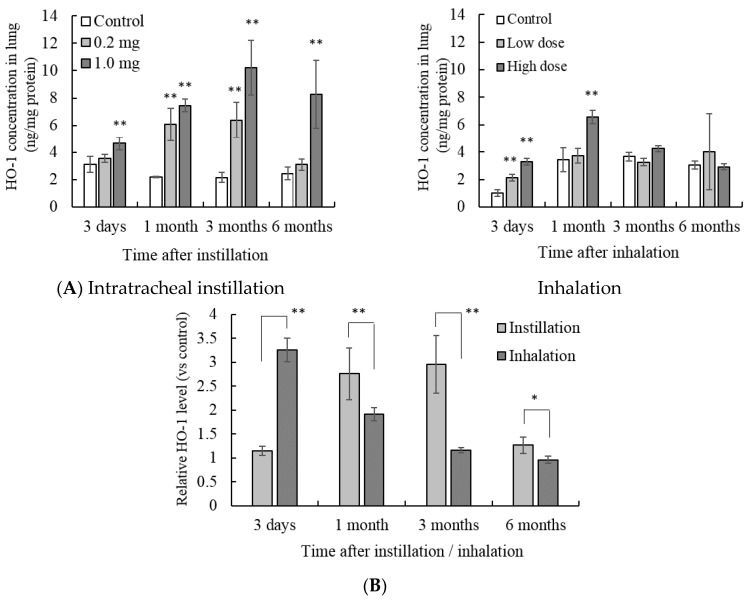
Influence of NiO nanoparticle exposure on the concentration of heme oxygenase-1 (HO-1) in lung tissue. (**A**) The concentration of HO-1 in the lung after intratracheal instillation and inhalation. The HO-1 concentration was measured by enzyme-linked immunosorbent assay (ELISA). Values are mean ± SE (*n* = 5). Significant differences *versus* control group are indicated in the figure (ANOVA, Dunnet). ** *p* < 0.01; (**B**) Comparison of 0.2 mg intratracheal instillation and high dose inhalation. The data show the relative level of the HO-1 protein concentration against each control group. ** *p* < 0.01, * *p* < 0.05 (ANOVA, Tukey).

There were no significant differences in body weights between the control animals and the NiO administrated animals through the experimental periods. The concentration of the oxidative stress response protein, heme oxygenase-1 (HO-1), in the lung tissue increased by both intratracheal instillation and inhalation of NiO nanoparticles (Figure 1A). The induction of HO-1 was dose dependent. HO-1 protein concentration in the 1.0 mg intratracheal instillation group was significantly higher than that in the untreated control group at 6 months after instillation.

There was no significant difference in HO-1 concentration between the 0.2 mg group and the control group at 6 months after instillation. With inhalation exposure, both the low and high dose showed an increase of HO-1 concentration in the lung 3 days after inhalation. Subsequently, there was no significant difference in HO-1 concentration between the inhalation group and the control group up to 3 months after inhalation. In these experiments, the amount of NiO nanoparticles in the lungs of the 0.2 mg instillation group and the high dose inhalation group was approximately the same [8]. The lung burden of NiO in both groups was approximately 134 μg at 3 days after instillation/inhalation. Thus, the HO-1 protein concentrations in the 0.2 mg intratracheal instillation and the high dose inhalation groups were compared (Figure 1B).

**Figure 2:**
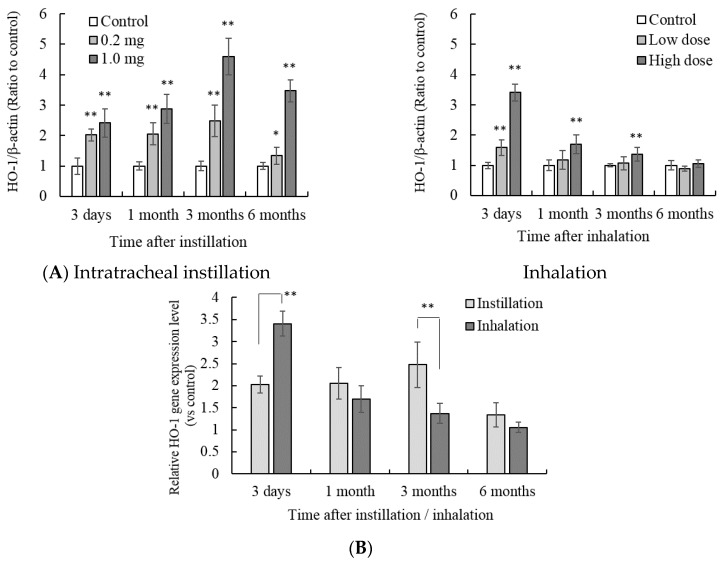
Influence of NiO nanoparticle exposure on the gene expression of HO-1 in the lung. (**A**) *ho-1* gene expression in the lung after intratracheal instillation and inhalation. The gene expression of *ho-1* was determined by real-time polymerase chain reaction (PCR). Values are mean ± SE (*n* = 5). Significant differences *versus* control group are indicated in the figure (ANOVA, Dunnet). ** *p* < 0.01, * *p* < 0.05; (**B**) Comparison of 0.2 mg intratracheal instillation and high dose inhalation. ** *p* < 0.01 (ANOVA, Tukey).

The value is indicated by a relative value in which the HO-1 concentration of each control group is 1. The peak of HO-1 concentration was different between intratracheal instillation and inhalation. The peak HO-1 concentration after intratracheal instillation was observed from 1 to 3 months after instillation. In contrast, inhalation of NiO nanoparticles showed a peak HO-1 concentration 3 days after inhalation, and subsequently the HO-1 concentration decreased with time. This trend was similar to that seen with the gene expression of HO-1 (Figure 2A). The gene expression of HO-1 increased 3 days after intratracheal instillation of 0.2 mg NiO nanoparticles. When the amount of NiO in the lung was similar, the peak of HO-1 gene expression was observed 3 months after intratracheal instillation, as opposed to 3 days after inhalation (Figure 2B).

The profile of the oxidative stress response enzyme, HO-1, differed after intratracheal instillation and inhalation at both the gene expression and protein level. Induction of the gene expression of HO-1 was observed 1 month and 3 months after intratracheal instillation. In contrast, HO-1 gene expression was already increased 3 days after inhalation. The gene expression of HO-1 after inhalation of NiO subsequently decreased with time. This difference between intratracheal instillation and inhalation might depend on the time necessary for the accumulation of NiO nanoparticles in the lungs. In this study, NiO nanoparticles were administrated to the lung by single intratracheal instillation. Specifically, the final lung burden of the NiO nanoparticles was almost similar in the intratracheal instillation and inhalation exposure. Therefore, even if the amount of NiO nanoparticles in the lung at 3 days after instillation/inhalation was similar, the pulmonary oxidative stress in inhalation exposure might be increased gradually for 1 month. Weak oxidative stress induces activation of anti-oxidative system [13]. While the NiO nanoparticles in the lung gradually increase after continuous inhalation, the anti-oxidative stress system might be induced. According to cellular experiments, exposure of NiO nanoparticles caused induction of HO-1 expression and then the cellular oxidative stress was reduced [9]. Therefore, oxidative stress in the inhalation group may be weaker than intratracheal instillation group in early period.

### 3.2. Concentration of Lipid Peroxide in the Lung Tissue

**Figure 3:**
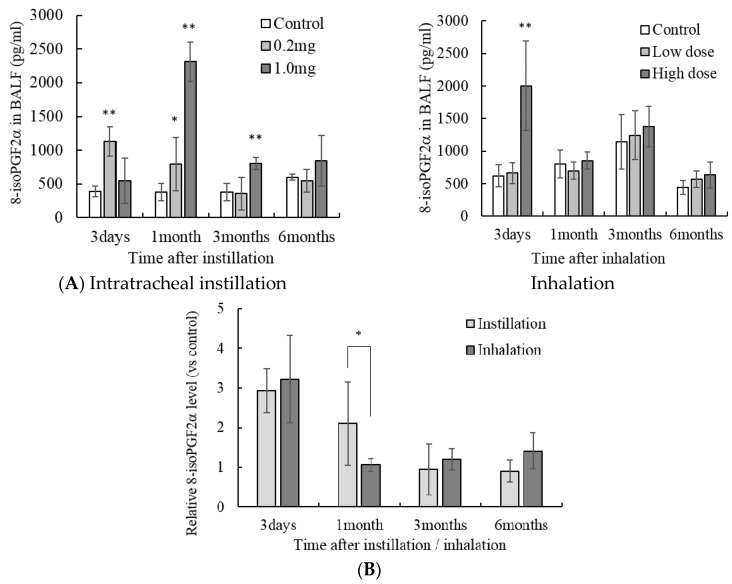
Influence of NiO nanoparticle exposure on the concentration of 8-iso-prostaglandin F2α in the bronchoalveolar lavage fluid (BALF). (**A**) The concentration of 8-iso-prostaglandin F2α in the BALF after intratracheal instillation and inhalation. The 8-iso-prostaglandin F2α concentration was measured using ELISA. Values are mean ± SE (*n* = 5). Significant differences *versus* control group are indicated in the figure (ANOVA, Dunnet). ** *p* < 0.01, * *p* < 0.05; (**B**) Comparison of 0.2 mg intratracheal instillation and high dose inhalation. The data show the relative level of the 8-iso-prostaglandin F2α concentration against each control group. * *p* < 0.05 (ANOVA, Tukey).

The concentration of 8-iso-PGF2α, which is an oxidation product of arachidonic acid in the lung, increased until 3 months after intratracheal instillation of 1.0 mg NiO nanoparticles (Figure 3A). In the 0.2 mg NiO nanoparticles injection group, 8-iso-PGF2α was increased until 1 month after instillation. Inhalation of NiO nanoparticles caused an increase of 8-iso-PGF2α in the lung only 3 days after a high dose of inhalation. Comparison of intratracheal instillation and inhalation showed little difference at 1 month. However, the tendency of 8-iso-PGF2α in the lung was similar between intratracheal instillation and inhalation (Figure 3B).

### 3.3. Concentration of Trx in the Lung Tissue

**Figure 4:**
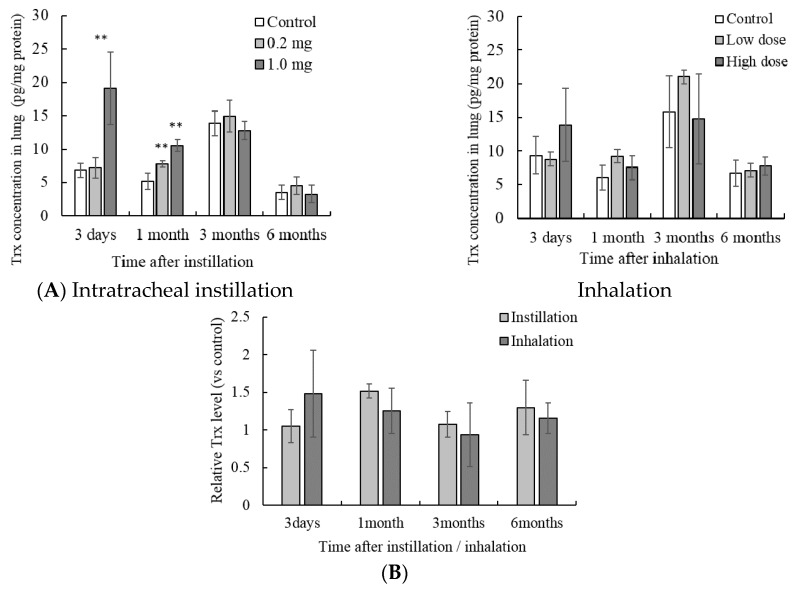
Influence of NiO nanoparticle exposure on the concentration of thioredoxin (Trx) in lung tissue. (**A**) The concentration of Trx in the lung after intratracheal instillation and inhalation. The Trx concentration was measured using ELISA. Values are mean ± SE (*n* = 5). Significant differences *versus* control group are indicated in the figure (ANOVA, Dunnet). ** *p* < 0.01; (**B**) Comparison of 0.2 mg intratracheal instillation and high dose inhalation. The data show the relative level of the Trx protein concentration against each control group.

The concentration of Trx in the lung tissue after 1.0 mg intratracheal instillation was higher than that in the control group after 3 days and 1 month (Figure 4A). Further, the concentration of Trx after 0.2 mg intratracheal instillation was higher after 1 month only. Inhalation of NiO nanoparticles did not show Trx induction at any time point. Comparison of intratracheal instillation and inhalation did not show significant differences (Figure 4B).

### 3.4. Gene Expression of Inducible Nitric Oxide Synthase (iNos) in the Lung Tissue

**Figure 5:**
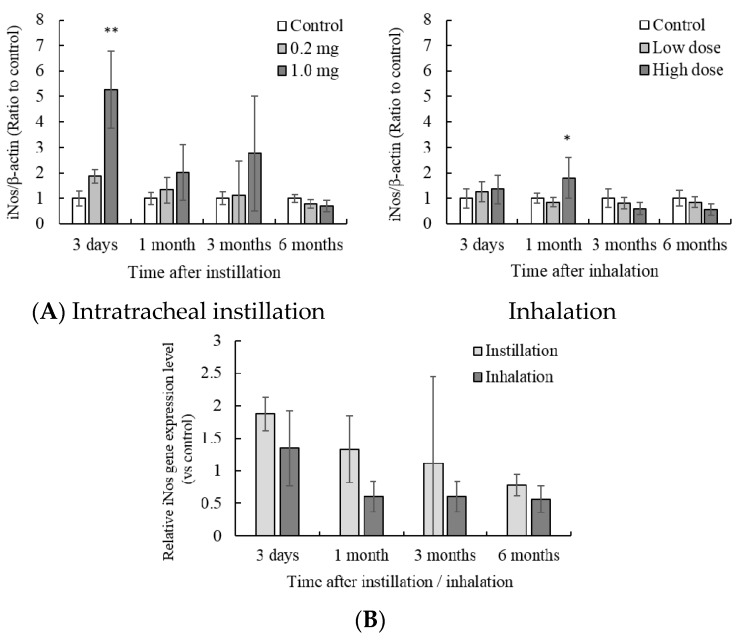
Influence of NiO nanoparticle exposure on the gene expression of iNos in the lung. (**A**) iNos gene expression in the lung after intratracheal instillation and inhalation. The gene expression of iNos was determined by real-time PCR. Values are mean ± SE (*n* = 5). Significant differences *versus* control group are indicated in the figure (ANOVA, Dunnet). ** *p* < 0.01, * *p* < 0.05; (**B**) Comparison of 0.2 mg intratracheal instillation and high dose inhalation.

Gene expression of iNos in the lung tissue was significantly higher than that in the control group 3 days after 1.0 mg intratracheal instillation or high dose inhalation (Figure 5A). Comparison of intratracheal instillation and inhalation did not show significant differences (Figure 5B).

### 3.5. Concentration of MPO in the Lung Tissue

**Figure 6:**
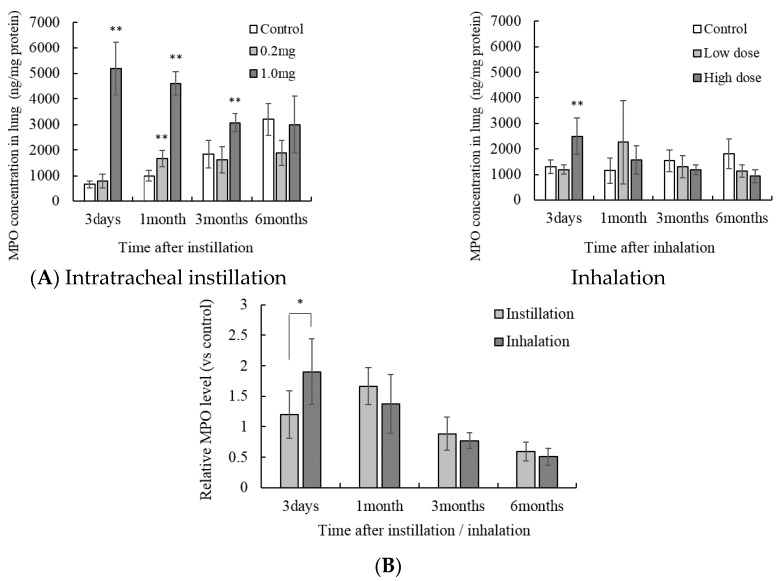
Influence of NiO nanoparticle exposure on the concentration of myeloperoxidase (MPO) in the lung tissue. (**A**) The concentration of MPO in the lung after intratracheal instillation and inhalation. The MPO concentration was measured using ELISA. Values are mean ± SE (*n* = 5). Significant differences *versus* control group are indicated in the figure (ANOVA, Dunnet). ** *p* < 0.01; (**B**) Comparison of 0.2 mg intratracheal instillation and high dose inhalation. The data show the relative level of MPO protein concentration against each control group. * *p* < 0.05 (ANOVA, Tukey).

The concentration of MPO in the lung increased until 3 months after 1.0 mg intratracheal instillation (Figure 6A). Further, the concentration of Trx was higher only 1 month after 0.2 mg intratracheal instillation. High dose NiO nanoparticle inhalation showed an increase of MPO 3 days after inhalation. Compared with that after intratracheal instillation, the MPO concentration was higher 3 days after inhalation (Figure 6B).

NiO nanoparticle-induced pulmonary oxidative stress can be divided into primary oxidative stress and secondary oxidative stress. Primary oxidative stress is caused by the nanoparticle itself. For example, some nanoparticles generate ROS on the surface. Secondary oxidative stress is caused by macrophages and neutrophils. These cells accumulate during inflammatory responses such as the secretion of chemokines and generate ROS [14]. We previously reported a comparison of inflammation responses caused by intratracheal instillation and inhalation of NiO nanoparticles [8].

According to the previous report, the number of neutrophils in the BALF was higher than that in the untreated control group at 1 month after intratracheal instillation of 0.2 mg of NiO nanoparticles. On the other hand, in the high dose NiO inhalation group, the neutrophils were significantly higher in the BALF than in the untreated control group at 3 days after inhalation. There were no significant differences between the inhalation group and control group 1 month after inhalation. These results corresponded to the observed MPO concentrations in the lung in this study. MPO catalyzes the reaction generating HOCl from H_2_O_2_ and Cl^−^ [15]. ROS are also generated during this reaction. These reactions not only induce a bactericidal effect and organic destruction, but also oxidative stress [16]. It is suggested that secondary oxidative stress is induced in the lung by MPO activity caused by the accumulation of neutrophils. After inhalation exposure, the accumulation of neutrophils and induction of secondary oxidative stress may be milder than that after a single intratracheal instillation.

### 3.6. Gene Expression of MT in the Lung Tissue

**Figure 7:**
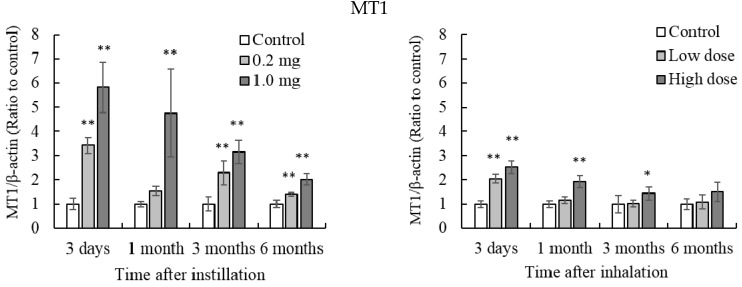

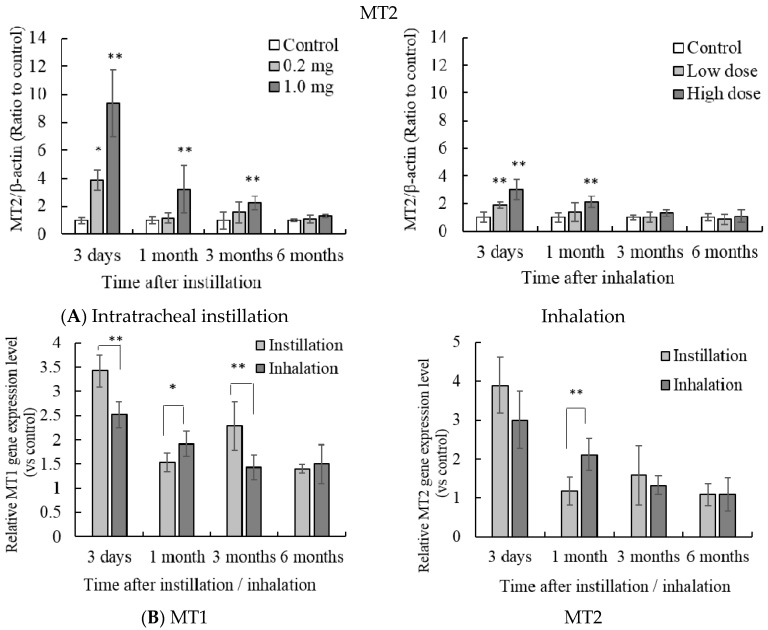
Influence of NiO nanoparticle exposure on the gene expression of metallothionein in the lung. (**A**) *mt1* and *mt2* gene expression in the lung after intratracheal instillation and inhalation. The gene expression of *mt* was determined by real-time PCR. Values are mean ± SE (*n* = 5). Significant differences *versus* control group are indicated in the figure (ANOVA, Dunnet). ** *p* < 0.01, * *p* < 0.05; (**B**) Comparison of 0.2 mg intratracheal instillation and high dose inhalation. ** *p* < 0.01, * *p* < 0.05 (ANOVA, Tukey).

Both intratracheal instillation of 0.2 mg and 1.0 mg of NiO nanoparticles caused induction of MT1 gene expression up until 6 months after instillation (Figure 7). Gene expression of MT2 increased at only 3 days and until 3 months after 0.2 mg and 1.0 mg intratracheal instillation, respectively. High dose NiO inhalation caused induction of MT1 gene expression for up to 3 months. Low dose NiO inhalation caused induction of MT1 gene expression 3 days after inhalation. Gene expression of MT2 was significantly increased until 1 month and 3 days after high-dose and low-dose NiO inhalation, respectively. Comparison of intratracheal instillation and inhalation did not show remarkable differences.

Induction of the *MT* gene suggests Ni^2+^ release from the NiO nanoparticles. Gene expression of MT is induced by metal ions via the metal response element (MRE)-binding transcription factor MTF [17].

However, on the other hand, expression of MT gene is also induced by an oxidation stress as well as metal ion. [17,18]. Examination of contribution of oxidative stress on the MT gene expression may be necessary. At least, gene expression profile of MTs suggested that Ni^2+^ and oxidative stress in lung was decreased with time.

## 4. Conclusions

**Figure 8:**
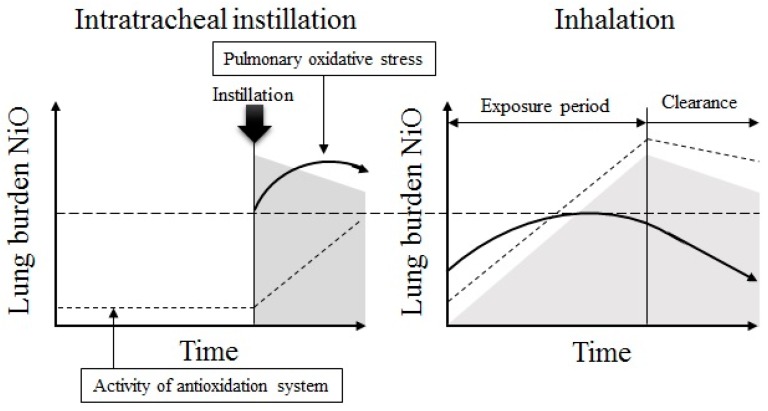
Estimated oxidative stress induction in intratracheal instillation and inhalation of NiO nanoparticles. If the amount of NiO in the lung is similar, the general pulmonary oxidative stress response is similar between intratracheal instillation and inhalation. The difference in oxidative stress strength depends on the induction of anti-oxidative system.

Intratracheal instillation and inhalation were conducted using the same NiO nanoparticles. Inflammation responses and lung injury and the amount of NiO in the lung caused by these examinations have been previously reported [8]. In particular, the retained amount of NiO nanoparticles was similar after intratracheal instillation (0.2 mg) and inhalation (high dose). The clearance of NiO nanoparticles from the lung after intratracheal instillation and inhalation was also the same. If the amount of NiO in the lung is similar, the general pulmonary oxidative stress response is similar between intratracheal instillation and inhalation. Both the intratracheal instillation and inhalation of NiO nanoparticles induce pulmonary oxidative stress. However, the state of pulmonary oxidative stress in the early phase was different between the two, even if the amount of NiO in the lung was similar. Single intratracheal instillation causes major pulmonary oxidative stress. In contrast, inhalation causes milder and continuous oxidative stress, which leads to induction of the anti-oxidative system (Figure 8). On evaluation of nanoparticle-induced pulmonary oxidative stress in the early phase, we should understand the difference between oxidative stress induced by intratracheal instillation and inhalation.
